# Removal of amoxicillin via chromatographic monolithic columns: comparison between batch and continuous fixed bed

**DOI:** 10.55730/1300-0527.3520

**Published:** 2022-11-29

**Authors:** Mustafa Deniz AĞLAMAZ, Koray ŞARKAYA, Deniz TÜRKMEN, Mustafa UÇAR, Adil DENİZLİ

**Affiliations:** 1 Department of Chemistry, Faculty of Science, Hacettepe University, Ankara, Turkey; 2 Department of Chemistry, Faculty of Science, Pamukkale University, Denizli, Turkey; 3 Department of Chemistry, Faculty of Science and Arts, Afyon Kocatepe University, Ankara, Turkey

**Keywords:** Monolithic column, hydrophobic interaction, liquid chromatography, amoxicillin, adsorption

## Abstract

This study presented a hydrophobic interaction-based poly(HEMA-MATrp) monolithic chromatographic column (MCC) to remove amoxicillin from aqueous solutions. In addition to their porous structure, monolithic-filled columns offer superior properties without loss of performance, which is one of the points that make them unique. The specific surface area of the monolithic column synthesized by the bulk polymerization of 2-hydroxyethyl methacrylate and N-Methacryloyl-L-tryptophan. Also, poly(HEMA-MATrp) MCC has been characterized via FTIR, SEM, and elemental analysis. According to BET analysis, the specific surface area of the poly(HEMA-MATrp) monolithic chromatographic column (MCC) is 14.2 mg/g. The adsorption and desorption of amoxicillin in an aqueous solution were investigated comparatively in both continuous fixed bed and batch adsorption. The highest adsorption value of amoxicillin was determined at pH 7 in the presence of PBS as 62.11 mg/g. The appropriate adsorption isotherm for the adsorption of amoxicillin was Langmuir, and the reaction kinetics was pseudo-second-order. No significant loss was observed for the adsorption capacity of poly(HEMA-MATrp) MCC after the 5 cycles of adsorption-desorption studies. Also, the loss for the adsorption capacity of the monolithic column is just %5.2 after 6-month storage, proving the reusability and storability of the monolithic column.

## 1. Introduction

Antibiotics are drugs of great importance for the continuation of health that produces to treat or prevent infectious diseases in humans and animals by preventing or controlling the growth of microorganisms such as bacteria, fungi, or protozoa [1]. However, pharmaceutical antibiotics have become an important class of pollutants in water resources as they enter water resources through discharges from pharmaceutical industries and wastewater plants [2]. Some antibiotics have high toxicity, low biodegradability, and are mutagenic in aquatic and terrestrial environments. Even carcinogenic properties [3] and their presence in water due to excessive consumption causes a decrease in drinking water quality [4], the proliferation of bacterial drug resistance, and increased concerns for their poor metabolism [5]. Amoxicillin (the empirical formula is C_16_H_19_N_3_O_5_S, or (2S,5R,6R)-6-[[(2R)-2-amino-2-(4-hydroxyphenyl)acetyl]amino]-3,3-dimethyl-7-oxo-4-thia-1 azabicyclo[3.2.0]heptane-2-carboxylic acid, as IUPAC name) which is among the B-lactam group antibiotics, is also a broad-spectrum penicillin derivative that contains a lactam ring and an aromatic group. These penicillins are drugs close to penicillin G against gram-positive and gram-negative cocci, with much higher efficacy than antistaphylococcal penicillins [6]. In addition, amoxicillin is used effectively to treat ear-nose-throat and urinary tract infections, sinusitis, bronchitis, and pneumonia [7]. Amoxicillin is one of the most common antibiotics used in modern medicine with significant ecotoxicity [8]. For instance, current assessments for measuring amoxicillin in industrial waste and drinking water for local sources have revealed that amoxicillin concentrations varied between ng/L and mg/L [9, 10]. Since its long existence and bioaccumulation in nature, there is concern that this class of antibiotic chemicals may alter the natural balance of ecosystems, causing toxic effects [11]. Because antibiotics are stable against biodegradation, common biotechnological wastewater treatment is insufficient to remove them [12][13]. Therefore, there is a need for effective and efficient methods for removing antibiotics from aqueous media. Various methods for removing or eliminating amoxicillin and similar antibiotics have been reported in the literature, some of these methods are such as membrane technology [14][15], sand filtration [16], chemical coagulation [17] or flocculation [18], chlorination [19], ultraviolet radiation [20], ozonation [21], and advanced oxidation processes [22], hybrid applications [23][24]. New approaches are still required in this field, though, as these are not regarded as ideal alternatives [25]. The adsorption method, which is easy to apply, practical and inexpensive, is thought to remove contaminants while treating wastewater [26]. Researchers in this field have used various materials with adsorbent properties such as natural minerals [27], carbon nanotubes [28], graphene oxide [29] or graphene hydrogels [30], humic acid [31] or activated carbon [32] in applications based on the adsorption method for removal of antibiotic derivatives as contaminants.

Monolithic stationary phases are one of the most effective microseparation techniques, designed as chromatographic support materials [33, 34]. Although conventional packed columns are used in many industrial processes, these systems’ efficiency and operating speed are adversely affected by factors such as slow mass transfer, large interparticle spaces, and irregularities against pressure changes [35]. The difference between monoliths and conventional packed columns is the monolith’s one-piece porous structure. Due to the interconnected porosity of the monoliths, the rapid mass transfer can be achieved through the interchannel networks formed. As the porosity increases for monoliths, large-structured biomolecules such as proteins are bioseparated with efficiency [36]. Monolithic columns can be evaluated under three groups in terms of their historical development as organic-polymer-based, inorganic silica-based, and organic-inorganic silica-based. Organic polymer-based monolithic columns have advantages such as simple polymerization conditions, application efficiency in a wide pH range, and easy modification of their surfaces by using functional monomers and crosslinkers [37] [38]. Hydrophobic interaction chromatography is based on separating and purifying biomolecules based on differences in their surface hydrophobicity. This method is used for industrial purification of antibodies, recombinant proteins, and products such as plasmid DNA, defining specific selectivity.

The aim of this study is the separation of amoxicillin from aqueous solutions by hydrophobic interaction. For this purpose, poly(HEMA-MATrp) based monolithic chromatographic column (MCC) was synthesized. This monolithic column was characterized via SEM, BET, and FTIR. Adsorption and desorption of amoxicillin from an aqueous solution through poly(HEMA-MATrp) MCC were studied. Also, the reusability of MCC was investigated.

## 2. Experimental

### 2.1. Materials

2-Hydroxyethyl methacrylate (HEMA), the main monomer for the synthesis of poly(HEMA-MATrp) monoliths, as well as Ethylene glycol dimethacrylate (EGDMA) were obtained from Fluka AG (Buchs, Switzerland). Amoxicillin was purchased from Sigma Chemical Co. (St. Louis, Missouri, USA). Potassium persulfate (KPS), potassium carbonate and all other chemicals used are of analytical grade and were obtained from Merck AG (Darmstadt, Germany). All monomers used in this study were stored at +4 °C. Ultradistilled water was used in the experiments.

### 2.2. Synthesis of MATrp

N-Methacryloyl-L-tryptophan (MATrp) monomer was synthesized as previously suggested in the literature [39]. According to this, 5 g of L-tryptophan and 2 g of NaNO_3_ have been added into 30 mL of 5% (w/v) K_2_CO_3_ solution. The solution was cooled to 0 °C. After this cooling process, 4 mL of methacryloyl chloride was added dropwise to the solution thoroughly with a nitrogen (N_2_) gas environment. The forming solution’s pH was 7. Then, this solution was extracted with ethyl acetate, and the liquid phase was removed under the evaporator. The remaining solid phase was crystallized diethyl ether and cyclohexane to obtain MATrp monomer.

### 2.3. Preparation of poly(HEMA-MATrp) MCC

For the synthesis of poly(HEMA-MATrp) monolithic column in a glass tube via bulk polymerization, 1 mL of 2-hydroxyethyl methacrylate (HEMA) was polymerized with N-Methacryloyl-L-tryptophan (MATrp) in the presence of ethylene glycol dimethacrylate as crosslinker reagent. The monolithic column was washed with distilled water and ethyl alcohol after polymerization to remove the impurities. The preparation conditions of poly(HEMA-MATrp) MCC are given in Table 1.

### 2.4. Characterisation studies of poly(HEMA-MATrp) MCC

#### 2.4.1. NMR analysis of MATrp

^13^C and ^1^H NMR spectrums of MATrp monomer were recorded in DMSO-d6 and D2O using with 500 MHz-Bruker Avance III 500 spectrometer (Bruker BioSpin GmbH, Rheinstetten, Germany) instrument. CHCl_3_ without deuterium was used as an internal reference. Chemical shifts (δ) with concentrations in ppm were evaluated against the internal reference.

#### 2.4.2. FTIR analysis of poly(HEMA-MATrp) MCC

The characteristic functional groups for poly(HEMA-MATrp) monolith were analysed by FTIR spectroscopy (FTIR 8000 Series, Shimadzu, Japan). Before analysis, monolith pieces were dried in an oven for 18 h. Three milligrams monolith was mixed with 97 mg KBr to homogenize, and this mixture was turned into pellets and made ready for FTIR analysis.

#### 2.4.3. Effect of back pressure and flow rate parameters

The flow rate-back pressure relationship was investigated on a poly(HEMA-MATrp) monolithic column. Back pressures at different flow rates (from 0.50 to 2.5 mL/min) were recorded and evaluated as data.

#### 2.4.4. Surface morphology and surface area measurements

Scanning Electron Microscopy (SEM) (JEOL, JEM 1200EX, Tokyo, Japan) showed the surface morphology of the poly(HEMA-MATrp) monolithic column. For this purpose, a thin surface section was taken from the synthesized monolith to take SEM images. The monolith surface was covered with a thin gold layer under vacuum, then readily recorded SEM images were. The surface area measurements of the poly(HEMA-MATrp) monoliths were performed with the Brunauer-Emmett-Teller (BET) instrument (Quantochrome SI, USA). Monoliths were weighed and placed in the sample measuring part of the device. It was kept in N_2_ for 11 h at 90 °C. Gas adsorption and desorption were carried out at −210 °C and 25 °C, respectively.

### 2.5. Elemental analysis

Elemental analysis was preferred for determining the MATrp content in poly(HEMA-MATrp) MCC. A dried monolith sample weighing 1 mg was placed in the device (Leco, CHNS-932, USA). The oxygen, carbon, hydrogen, and nitrogen contents in the sample were determined by combustion.

### 2.6. Adsorption-desorption studies

#### 2.6.1. Adsorption of amoxicillin from aqueous solutions

The adsorption of amoxicillin from aqueous solutions in the batch system to poly(HEMA-MATrp) monolith was investigated. For monolithic poly(HEMA-MATrp) columns, the effects of changes in amoxicillin concentration (10–500 mg/mL) and temperature (4–40 °C) on adsorption capacity and rate were investigated at pH= 7. Spectrophotometric determination of amoxicillin was measured at λ= 230 nm in UV-spectrophotometry. The interaction between the functional ligand MATrp and amoxicillin was evaluated in kinetic, isotherm model, and thermodynamic data. The amount of amoxicillin (qa) adsorbed at equilibrium was determined with the following equation (Eq. 1):


(1) $$q_{a}=\frac{\left(c_{i}-c_{f}\right)}{m}$$

In this relation, *q_a_* is the amount of amoxicillin adsorbed by the poly(HEMA-MATrp) MCC (mg/g); *c_i_* is the initial concentration (mg/L) of amoxicillin; *c_f_* is the equilibrium concentration of amoxicillin after adsorption (mg/L); m is the mass of poly(HEMA-MATrp) MCC (g), and V is the volume of solution (L).

#### 2.6.2. Comparison between batch and continuous fixed bed adsorption

The adsorption of amoxicillin from an aqueous solution on a poly(HEMA-MATrp) MCC was investigated in both batch and continuous systems. In this context, experimental conditions for a 100 mg/L concentration of amoxicillin solution were adjusted at pH 7 and temperature at 25 °C. All monoliths in the column were turned into powder and used in the batch system for adsorption studies. Powdered monoliths were placed into amoxicillin solution and kept on the rotator for different times (between 0 min and 60 min). Finally, the particles were precipitated by centrifugation, and the adsorbed amoxicillin values were recorded spectrophotometrically.

In another attempt, the solution was passed through a poly(HEMA-MATrp) column with a 0.5 mL/min flow rate for the continuous fixed bed system at different times (between 0 min and 60 min). Spectrophotometric analysis was also used to determine antibiotic concentration.

#### 2.6.3. Desorption studies for reusability and storability

1 M Glycine hydrochloride solution was used as the desorption agent for amoxicillin adsorbed by poly(HEMA-MATrp). 1.0 M glycine hydrochloride solution was passed through amoxicillin-loaded poly(HEMA-MATrp) MCC with a 0.5 mL/min flow rate at room temperature. The amount of amoxicillin desorbed from the monolithic column was calculated using Eq. 2.


(2) $$\text{Soption}\%=\frac{\text{Concentration of amoxicillin released into the desorption medium}}{\text{Concentration of amoxicillin adsorbed by MCC}}\times 100$$

Poly(HEMA-MATrp) MCC was subjected to adsorption-desorption cycles as 5 times for the feasibility studies. The experimental procedure was applied in the same way. Poly(HEMA-MATrp) MCC was washed with 50 mM NaOH and deionized water for regeneration and cleaning. Also, long-term storage stability was explored by prestoring a poly(HEMA-MATrp) monolithic column in PBS (0.1 M, pH 7.4) at 4 °C for 6 months, and adsorption capacity was measured at various time intervals.

## 3. Results and discussion

### 3.1. Characterisation of MATrp monomer

The structural information of the MATrp monomer was characterized by both FTIR and NMR spectroscopies. The determination of the functional groups of MATrp was highlighted by the FTIR spectroscopy method. Accordingly, the 3403 cm^−1^ (NH-stretching), 1585 cm^−1^ (the CO stretching of the amide), 1798 cm^−^1 (CO- stretching from the acid group), 1456 cm^−1^ (CN stretching of the aromatic ring), 1514 cm^−1^ (NH-bending), 1229 cm^−1^ (acid-induced CO-bending) and 1155 cm^−^1 (aromatic ring stretching) are shown in the FTIR spectrum. The characteristic peaks for the ^1^H-NMR spectra were determined as follows: 2H, 10 CH_2_ 3.42-3.36 (q), 3H, 17 CH_3_ 1.80 (s), 1H, 16 CH_2_ 5.33 (s), 1H, 11 CH 4.50–4.51 (m), 1H, 16 CH_2_ 5.64 (s), 1H, 12 OH (asit) 11.1 (s), 5H, 4,5,6,7,2 indol 7.15–7.57 (m), 1H,13NH (amid) 7.15 (d), 1H, 1NH (indol) 8.25 (d). The characteristic peaks for the 13C-NMR spectra were examined as follows: C (vinyl), 110.7 ppm; CH, 53.7 ppm; CH_2_, 65.3 ppm; CH_3_, 18.8 ppm; CH (indole), 111.9 ppm; CH (benzene ring), 118.9 ppm; CH (benzene ring), 121.36 ppm; C (indole), 127.7 ppm; C=O (amide), 167.9 ppm; CH (benzene ring), 118.7 ppm; CH (benzene ring), 139.9 ppm; CH_2_ (vinyl), 120.1 ppm; CH (benzene ring), 124.0 ppm; C=O (acid) is 173.8 ppm and C (benzene ring) is 136.6 ppm. FTIR, ^1^H-NMR and ^13^C-NMR spectrums of MATrp functional monomer can be accessed from the supplementary materials section.

### 3.2. Characterisation of poly(HEMA-MATrp) MCC

**Figure 1:**
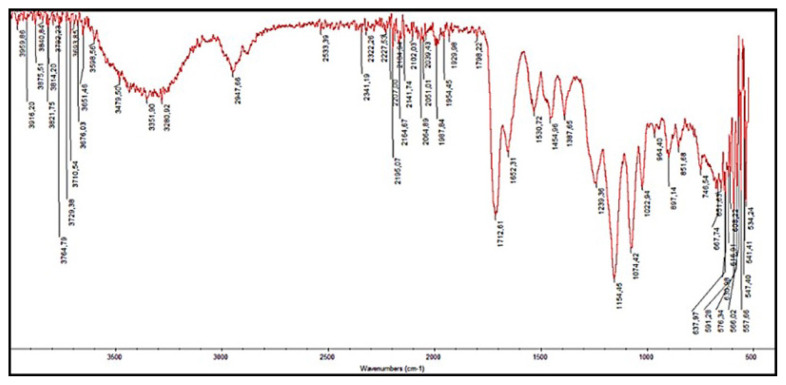
FTIR spectrum of poly(HEMA-MATrp) MCC.

In the synthesis part of this study, firstly, N-methacryloyl-L-tryptophan (MATrp) was synthesized as a pseudo-specific ligand. Then, the main monomer HEMA and MATrp formed poly(HEMA-MATrp) monolithic columns via the bulk polymerization method. FTIR spectrum of poly(HEMA-MATrp) is presented in Figure 1. According to the FTIR spectrum of poly(HEMA-MATrp) monolith, -OH stretching at 3350 cm^−1^, -CH stretching for aliphatic alkyl at 2947 cm^−1^, -C=O stretching at 1712 cm^−1^, -C=C stretching at 1652 cm^−1^, the –C-N stretch of the amide at 1454 cm^−1^ and 1387 cm^−1^, and at 1154 cm^−1^ peaks for the stretching of the aromatic ring are observed.

### 3.3. Investigation of correlation between backpressure and flow rate

**Figure 2:**
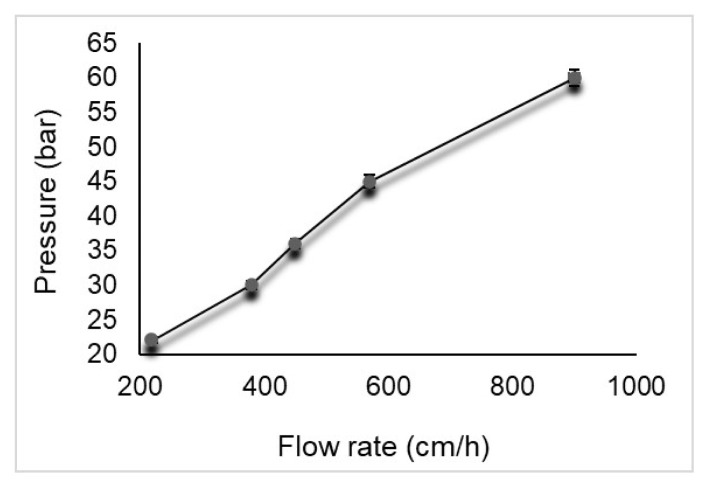
Correlation between backpressure flow rate.

Passing through the column was investigated at values ranging from 0.5 to 2.5 mL/min for examining the flow dynamics of the poly(HEMA-MATrp) monolith, and the flow rate of the solution. Figure 2 shows the correlation between backpressure and flow rate. Backpressure increases linearly with increasing flow rate for poly(HEMA-MATrp) monolith. Although the backpressure increases at high flow rates, this pressure is at values that will not force a standard HPLC system pump and system, thanks to the porous structure of the monolithic column. This point shows us that analysis can be performed very easily with poly(HEMA-MATrp) monolith at high flows and it will not affect the chromatographic system.

### 3.4. Surface morphology and surface area properties

**Figure 3:**
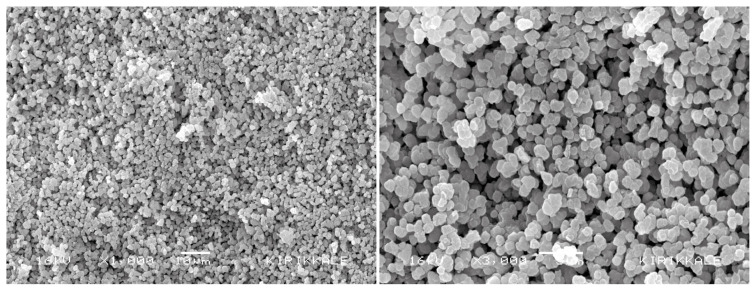
SEM photographs of poly(HEMA-MATrp) MCC.

The surface morphology of poly(HEMA-MATrp) monolithic columns was investigated using SEM. SEM images of the monolithic column have shown that monoliths have relatively narrow pores but a very rough structure, as seen in Figure 3. The monolith of poly(HEMA-MATrp) has a considerable surface area due to its rough structure. On the other hand, the surface area of the monolith, of which HEMA and MATrp are the main components, was determined as 14.2 m^2^/g. Thanks to the surface area of the monolithic column, the mass transfer of amoxicillin from an aqueous solution is faster and easier, thus increasing the adsorption capacity.

### 3.5. Elemental analysis of poly(HEMA-MATrp)

Elemental analysis was performed to determine the amount of MATrp added to the content of the poly(HEMA-MATrp)-MCC. The amount of MATrp was calculated based on nitrogen, using stoichiometric ratios as a result of combustion. Accordingly, the amount of MATrp was calculated as 375 μmol/g monoliths. The only N_2_ source in the monolithic column must be the functional monomer (MATrp). Due to its structure, HEMA does not contain nitrogen.

### 3.6. Adsorption studies

#### 3.6.1. Effect of concentration

**Figure 4:**
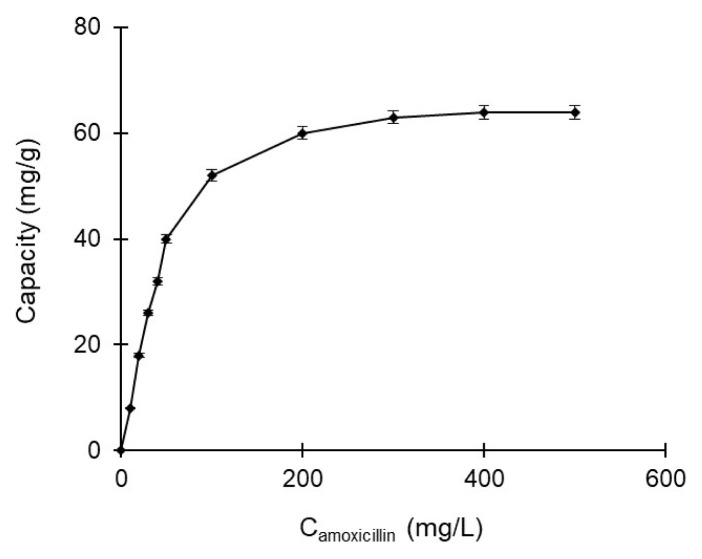
The effect of amoxicillin concentration for amoxicillin adsorption with poly(HEMA-MATrp) MCC; pH 7.0, T: 25 °C, flow rate: 0.5 mL/min, t: 60 min.

The effect of amoxicillin concentration on the adsorption of amoxicillin through with poly(HEM-MATrp) monolith column was investigated. In this context, amoxicillin solutions at concentrations ranging from 0 to 500 mg/L were passed through the monolithic column, and the adsorption values are shown in Figure 4. The graph represents the effect of the initial concentration of amoxicillin on the adsorption relationship between amoxicillin and the monolithic column. As can be seen from the results, the equilibrium value was reached after 300 mg/L amoxicillin concentration. This result shows that amoxicillin fully interacts with the active specific ligands in the medium, and there is no point in interacting after this concentration.

#### 3.6.2. Effect of flow rate

**Figure 5:**
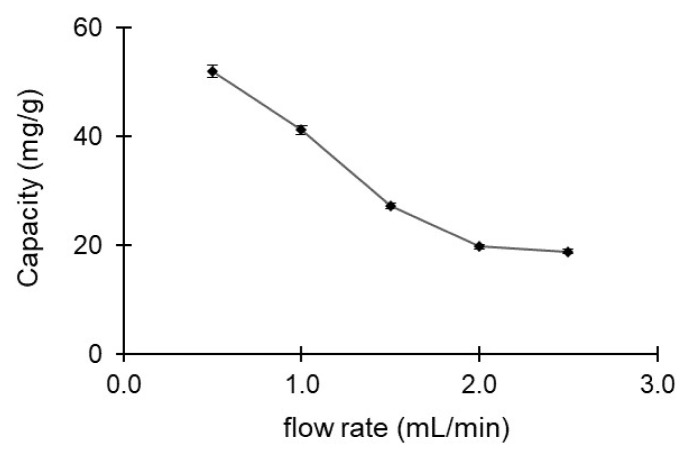
The effect of flow rate for amoxicillin adsorption with poly(HEMA-MATrp) MCC; pH 7.0, T: 25 °C, C_amoxicillin_: 100 mg/mL, t: 60 min.

The effect of flow rate for adsorption of amoxicillin from aqueous solution through by poly(HEMA-MATrp) monolithic column was investigated. At this part, the concentration of 100 mg/L amoxicillin was passed through a monolithic column at about flow rate of 0.5–2.0 mL/min, and the change of adsorption values was investigated. The effect of the difference in flow rates on the adsorption capacity of amoxicillin is shown in Figure 5. The adsorption capacity of amoxicillin decreases from 52.0 mg/g at a flow rate of 0.5 mL/min to 18.9 mg/g at a flow rate of 2.5 mL/min. The increasing flow rate decreases the time that amoxicillin molecules can interact with the MATrp functional group in the monolithic column. Since the interaction time between the analyte and the column decreased, also the retention time of amoxicillin in the monolithic column decreased with the flow rate increase.

#### 3.6.3. Effect of temperature

**Figure 6:**
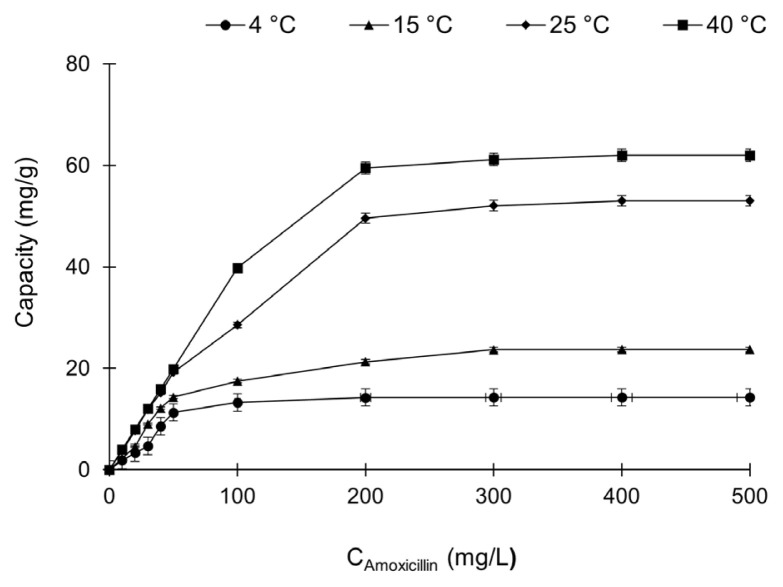
The effect of temperature for amoxicillin adsorption with poly(HEMA-MATrp) MCC at different concentrations of amoxicillin; pH 7.0, T: 4–40 °C, C_amoxicillin_: 0–500 mg/mL, flow rate: 0.5 mL/min, t: 60 min.

Adsorption results of amoxicillin solutions between 0 mg/L and 500 mg/L concentration value with poly(HEMA-MATrp) MCC at different temperatures were investigated. As seen in Figure 6, the adsorption values of amoxicillin tend to increase with increasing temperature. The temperature effect is a critical parameter to reveal the interaction based on adsorption. If ionic and hydrogen bonds are dominant interactions, changes in adsorption capacity are inversely proportional to temperature [40]. In contrast, if hydrophobic interactions and van der Waals forces are dominant interactions, changes in adsorption capacity are directly proportional to temperature [41]. In this context, it is expected that the adsorption of amoxicillin from aqueous solutions through hydrophobic poly(HEMA-MATrp) MCC increases with increasing temperature, considering the hydrophobic interactions.

#### 3.6.4. Comparison between batch and continuous fixed bed adsorption for amoxicillin removal

**Figure 7:**
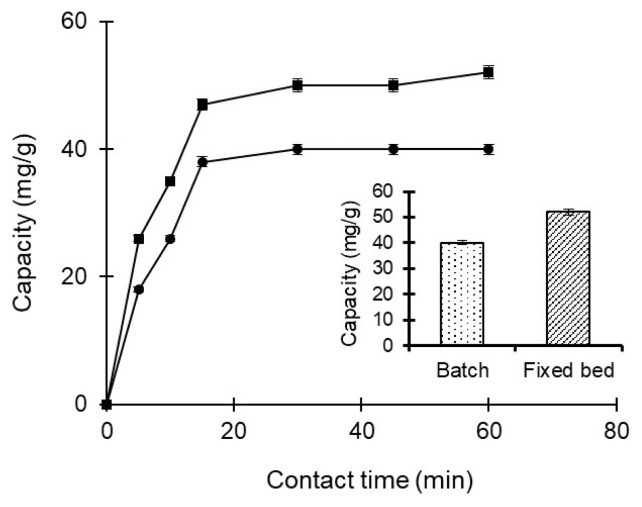
Comparative investigation of amoxicillin adsorption capacity through poly(HEMA-MATrp) MCC in batch and continuous fixed bed systems; pH 7.0, T: 25 °C, C_amoxicillin_: 100 mg/mL, t: 60 min.

The capacity values of amoxicillin adsorption from aqueous solutions occurring at different times between batch and continuous fixed bed systems were evaluated comparatively. As a result of comparing the total adsorption capacities (in Figure 7), the adsorption capacity via the continuous fixed bed system was found to be higher each time compared to the batch system. It is considered that a continuous system supports the direct mass transfer.

### 3.7. Adsorption thermodynamics

Parameters such as temperature change and amoxicillin concentration data were examined for the investigation of the thermodynamic correlation between poly(HEMA-MATrp) and amoxicillin in adsorption studies; thermodynamic values of ΔG, ΔH, and ΔS can be calculated using equilibrium constants that can change with temperature. The equilibrium constant as a function of temperature concerning the enthalpy change in adsorption is defined by the following equation (Eq. 3):


(3) $$\frac{d\;ln\;b}{dT}=\frac{\Delta H}{\Delta T^{2}}$$

In this equation; ΔH is the enthalpy of adsorption (j/mole), R is the gas constant (J/mole K), and b is the Langmuir constant. Depending on whether the ΔH value is positive or negative, the interaction of Langmuir constant (b) with temperature is examined. The sign of ΔH will be positive if endothermic adsorption occurs, and the sign of ΔH will be negative if an exothermic reaction occurs. In cases with positive signs, the Langmuir constant will increase with temperature. In cases with negative signs, the Langmuir constant will shift in the direction of decrease with increasing temperature.

Gibbs energy (G) is used, which is related to using the energy of a chemical reaction for work. The Gibbs energy (ΔG) of the reaction is given by the following equation (Eq.4):


(4) ΔG=ΔH-TΔS

Since temperature affects Langmuir constant (b) and Gibbs energy (ΔG), it can be expressed as following equations:


(5) ΔG=-RT ln b

(6) $$ln\;b=\frac{\Delta S}{R}+\frac{\Delta H}{RT}$$

The plot of lnb – 1/T for amoxicillin adsorption with the slope and intercept gives the values of “ΔH” “ΔS” and values, respectively [42].

According to the Van’t Hoff equation expressed by the following equation (Eq.7);


(7) $$ln\;K_{eq}=\frac{-\Delta H}{RT}+\frac{\Delta S}{R}$$

In the plot of lnKeq – 1/T; slope = - ΔH/R, and intercept = ΔS/R.

According to the Van’t Hoff equation, ΔH= 0.833 J/mole at 25 °C. The positive value of ΔH indicates that adsorption operates in an endothermic process. Again at the same temperature, ΔS= −89.68 J/mole K was calculated. ΔG was calculated as 26.724 kJ/mole K. Adsorption occurs with an endergonic system within these data. In other words, it is a system that requires external energy.

### 3.8. Adsorption isotherms

The adsorption mechanism can be analysed with the contributions of physicochemical parameters, thermodynamic assumptions, surface properties and affinity degree of adsorbents. The most commonly useful isotherms describing the adsorption process are the Freundlich and Langmuir isotherms.

The Langmuir isotherm model is defined with the following equation (Eq.8):


(8) $$\frac{C_{eq}}{Q}=\frac{1}{Q_{max}\frac{C_{eq}}{Q_{max}}}$$

In this equation; Q_max_ (mg/g) is the theoretical maximum amount of adsorbed amoxicillin, b (mL/mg) is the Langmuir constant related to the affinity of the binding sites, C_eq_(mg/mL) is the amount of amoxicillin remaining in the solution after adsorption, Q (mg/g) is adsorbed amoxicillin on the poly(HEMA-MATrp) MCC gives the amount of adsorbate.

The plot of 1/Q versus 1/C_eq_ gives 1/Q_max_ as intercept and 1/(Q_max_ b) as slope. The correlation coefficient (R^2^) calculated from the data from the adsorption results at different temperature values is high for all four different systems. These linear results show that adsorption occurs by the Langmuir isotherm.

Another model, which is the most traditional of the isotherms explaining the adsorption equilibrium, is the Freundlich adsorption model. Freundlich isotherm is a different form of the Langmuir isotherm and is applying to describe adsorption on the inhomogeneous surface. Freundlich model assumes that the adsorption energy changes depending on whether the neighbouring binding points connect or not, and it is expressed by the following equation (Eq. 9):


(9) $$Q_{eq}=K_{f}\times {C_{eq}}^{1/n}$$

This equation shows the adsorption amount Qeq (mg/g), C_eq_ amoxicillin concentration in the solution, K_f_ and n are Freundlich constants. As a result of taking the logarithm of both sides of the equation, the following equation is formed:


(10) $$ln\;Q_{eq}=ln\;K_{f}+\left(\frac{1}{n}\right)\times C_{eq}$$

According to the adsorption results based on the Freundlich model, the correlation coefficient is lower than the correlation coefficients in the Langmuir model results. For this reason, it is not concluded that adsorption takes place on a homogeneous surface, and accordingly, multilayer adsorption occurs. In addition, adsorption occurred in a single layer. It is determined that the Langmuir model is more suitable than the Freundlich model for this studies for the removal of amoxicillin from aqueous solution through poly(HEMA-MATrp) MCC.

The adsorption constants and correlation coefficients related to Langmuir and Freundlich models for amoxicillin adsorption from aqueous solution are presented in Table 2.

### 3.9. Adsorption kinetics

Pseudo-first-order and pseudo-second-order kinetic models are widely applied kinetic equations to describe adsorption systems and evaluate control parameters; therefore, they were used for adsorption kinetics of amoxicillin for this study. The experimental comparison of amoxicillin adsorption capacities and the theoretical values calculated via these two models are presented in Table 3.

The experimental values and the theoretical values of Q_e_ obtained from the pseudo-second-order kinetic equation show similarities. In addition, the correlation coefficient is high, and the R^2^ values are close to 1 according to the adsorption results observed at different concentrations. This indicates that the adsorption of amoxicillin via poly(HEMA-MATrp) MCC may be more suitable for the second-order kinetic model. According to these results, diffusion limitations in the adsorption of amoxicillin with monolith are negligible. The fact that the porous structure of the monolith allows a smooth flow over the amoxicillin solution can be shown as an explanation for this situation.

### 3.10. Reusability

**Figure 8:**
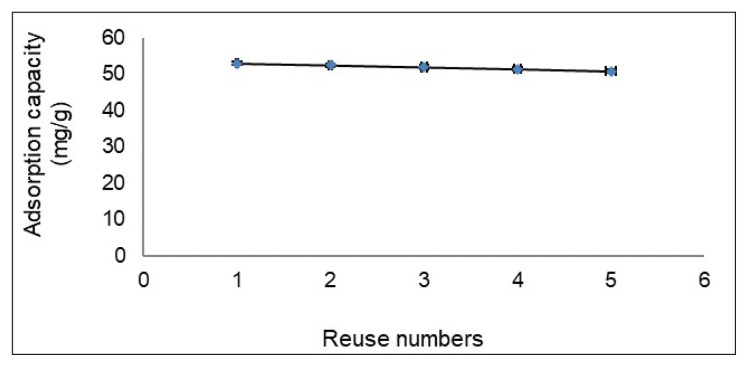
Reusability of poly(HEMA-MATrp) MCC; pH 6.0, T: 25 °C, C_amoxicillin_: 100 mg/mL, t: 60 min, flow rate: 0.5 mL/min.

Amoxicillin desorption was carried out in a continuous fixed-bed system using a 1.0 M glycine hydrochloride solution. Adsorption and desorption processes were repeated 5 times on pol(HEMA-MATrp) MCC, and results of repetitions are shown in Figure 8. As a result of repetitions, no significant loss in adsorption capacity was observed. For example, at the end of the 5th iteration, the value of the adsorption capacity is 50.8 mg/g due to only a 4.2% decrease. It is concluded that it is appropriate to use glycine hydrochloride as a desorption agent in this system. On the other hand, it appears that poly(HEMA-MATrp) MCC can be used for efficient adsorption repeats due to desorptions.

**Figure 9:**
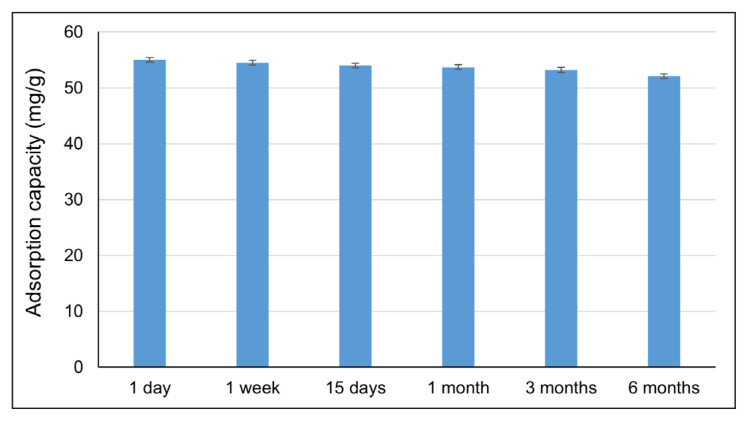
Storability of poly(HEMA-MATrp) MCC; pH 6.0, T: 25 °C, C_amoxicillin_: 100 mg/mL, t: 60 min, flow rate: 0.5 mL/min.

The poly(HEMA-MATrp) monolithic column was stored at 4 °C for 6 months. The values obtained from the adsorption processes are shown in Figure 9. According to the results, the adsorption process performed at various time intervals for storability of poly(HEMA-MATrp) was not significantly affected. Namely, while the adsorption capacity is 55.0 mg/g at the end of the first day, the loss is only 5.7% at the six months. These results highlight that the poly(HEMA-MATrp) monolithic column is stable in reusability and lifetime.

### 3.11. Comparison with other studies in the literature

There are studies aiming to remove amoxicillin from wastewater by using different adsorbents. In a study using organobentonites used as absorbents to remove amoxicillin from wastewater, it was reported that bentonite modified with hexadecyl trimethyl ammonium effectively removed amoxicillin from aqueous solution (Langmuir isotherm had the highest adsorption capacity (26.18 mg/g at 20 °C) noted.) [43]. Franco et al. studied the removal of amoxicillin from an aqueous solution by adsorption on activated carbon. They suggested that the adsorption of amoxicillin occurs predominantly by chemical adsorption, showing a maximum monolayer adsorption capacity of 4.4 mg/g [44]. Ali et al. used activated carbon modified with iron nanoparticles for removal of amoxicillin from aqueous solutions. It was reported that q_max_ was 40.282 mg/g as max adsorption capacity [45]. Chaba et al. prepared zinc oxide coated carbon nanofiber composite to use for the removal of amoxicillin from environmental media. According to the data, the greatest adsorption capacity was 156 mg/g [46]. In another study, NH_4_Cl-induced activated carbon was used for the removal of amoxicillin antibiotic from water. The maximum adsorption capacity of amoxicillin onto activated-carbon was 262.0 mg/g [47]. Caravaca et al. reported that 93% adsorption efficiency was reached with using of magnetic core nanoparticles functionalized with silver nanoparticles [48]. These results show that the performance of poly(HEMA-MATrp) monolithic chromatographic column (MCC) on adsorption of amoxicillin from aqueous solutions is comparable against other adsorbents in the literature so far.

## 4. Conclusion

In this study, a poly(HEMA-MATrp) monolithic column was prepared to use for the removal of amoxicillin from an aqueous solution. It was characterised by SEM, BET, FTIR, and elemental analysis methods. Adsorption studies of amoxicillin through poly(HEMA-MATrp) MCC were investigated both batch and continuous fixed-bed systems, and the maximum adsorption capacity was calculated as 62.11 mg/g at pH 7. Also, no significant loss in adsorption capacity was observed after adsorption-desorption studies for reusability of poly(HEMA-MATrp) MCC with storability after 6-month. As a result of the adsorption isotherm and kinetics analyses, the Langmuir isotherm model, whose correlation coefficient was calculated as 0.997, and the pseudo-second-order, since the experimental and theoretical Qeq values are very close to each other, seemed appropriate for this study. As a result, the new generation poly(HEMA-MATrp) monolithic chromatographic column, designed as an alternative to traditional particle-filled columns, has been used efficiently to remove amoxicillin from an aqueous solution with the contributions of hydrophobic interaction chromatography as well as a filler containing pseudo-specific ligand.

## Figures and Tables

**Table 1:** Preparation conditions of poly(HEMA-MATrp) MCC.

Column	HEMA (mL)	MATrp (in HEPES, 0.9%) (mg)	Toluene (mL)	Time (h)	Temperature (°C)
**poly(HEMA-MATrp)**	1.0	100.0	0.5	4	75

**Table 2:** Comparison of Langmuir and Freundlich Adsorption constants and correlation coefficients for amoxicillin adsorption.

Temperature	Freundlich isotherm			Langmuir isotherm			
(°C)	K_f_, mL/g	n	R^2^	Q_max_	b, L/mg	K_d_, mg/L	R^2^
**4**	1.75	2.57	0.71	15.24	0.04	24.23	0.993
**15**	3.09	2.68	0.70	25.19	0.04	23.60	0.994
**25**	10.41	3.28	0.83	54.05	0.13	7.56	0.997
**40**	20.51	4.37	0.82	62.11	1.32	0.76	0.996

**Table 3:** Pseudo-first-order and pseudo-second-order kinetic constants of amoxicillin adsorption.

Initial concentrations	Q_e_	Pseudo-first order			Pseudo-second order		
(mg/L)	(mg/g)	k_1_ (1/min)	Q_e_ (mg/g)	R^2^	k_2_ (1/min)	Q_e_(mg/g)	R^2^
20	18	0.0806	12.68	0.993	0.736	15.15	0.993
20	40	0.1151	36.56	0.979	0.066	38.60	0.972
100	53	0.1013	42.46	0.922	0.193	58.82	0.996
300	63	0.1404	61.23	0.919	0.179	71.43	0.992

## Data Availability

The authors can confirm that all relevant data are included in the article.
